# Equity in reproductive and maternal health services in Bangladesh

**DOI:** 10.1186/1475-9276-12-90

**Published:** 2013-11-14

**Authors:** Eyob Zere, Yuki Suehiro, Aminul Arifeen, Loshan Moonesinghe, Sanchoy K Chanda, Joses M Kirigia

**Affiliations:** 1 Department of Health Systems Financing, Health Authority, Abu Dhabi, United Arab Emirates; 2 United Nations Population Fund, Dhaka, Bangladesh; 3 World Health Organization, Regional Office for Africa, Brazzaville, Congo

## Abstract

**Background:**

The target date for achieving the Millennium Development Goals (MDGs) is now closer than ever. There is lack of sufficient progress in achieving the MDG targets in many low- and middle-income countries. Furthermore, there has also been concerns about wide spread inequity among those that are on track to achieve the health-related MDGs. Bangladesh has made a notable progress towards achieving the MDG 5 targets. It is, however, important to assess if this is an inclusive and equitable progress, as inequitable progress may not lead to sustainable health outcomes. The objective of this study is to assess the magnitude of inequities in reproductive and maternal health services in Bangladesh and propose relevant recommendations for decision making.

**Methods:**

The 2007 Bangladesh demographic and health survey data is analyzed for inequities in selected maternal and reproductive health interventions using the slope and relative indices of inequality.

**Results:**

The analysis indicates that there are significant wealth-related inequalities favouring the wealthiest of society in many of the indicators considered. Antenatal care (at least 4 visits), antenatal care by trained providers such as doctors and nurses, content of antenatal care, skilled birth attendance, delivery in health facility and delivery by caesarean section all manifest inequities against the least wealthy. There are no wealth-related inequalities in the use of modern contraception. In contrast, less desired interventions such as delivery by untrained providers and home delivery show wealth-related inequalities in favour of the poor.

**Conclusions:**

For an inclusive and sustainable improvement in maternal and reproductive health outcomes and achievement of MDG 5 targets, it essential to address inequities in maternal and reproductive health interventions. Under the government’s stewardship, all stakeholders should accord priority to tackling wealth-related inequalities in maternal and reproductive health services by implementing equity-promoting measures both within and outside the health sector.

## Background

The target date for achievement of the Millennium Development Goals (MDGs), 2015, is now only two years away. A few low- and middle-income countries have made commendable strides and are on track to achieving the health-related goals, mainly MDGs 4, 5 and 6. However, the majority of low- and middle-income countries are off-track and may not be able to achieve them in the remaining years without a redoubled effort [1].

Achievements in terms of population averages, however, do not often represent the condition of all socio-economic groups in a country. Evidence indicates that even in countries that are performing well, the mortality and morbidity burden of a section of the population, especially the poor and marginalized, has not improved [2]. A criticism leveled at the MDGs is the lack of explicit equity focus. The preoccupation has been on aggregate achievement [3]. The MDG indicators are worse off among the poor, rural areas and less educated segment of the population than otherwise.

Most of the health related MDG targets fail to address the issue of distribution of the burden of morbidity or mortality. For example, the goal of reducing the maternal mortality ratio by 75% between 1990 and 2015 can be achieved by a disproportionate improvement in the burden of maternal mortality among the well-off segment of society, while the burden on the poor may remain the same or even increase. Equity-focused approaches accelerate progress towards achieving the health-related MDGs faster and in a more cost-effective and sustainable manner [4]. Achieving the health-related MDGs without addressing inequities in health care and other determinants of health is difficult. It is among the poorest and worse-off groups of society that the indicators are unfavourable and there is a great potential for improvement if the situation of these disadvantaged groups improves [5].

Cognizant of this fact, the drive for a greater focus on equity in human development has gathered momentum at the global level [6,7]. The United Nations Commission on information and accountability for Women’s and Children’s Health places equity as one of the cornerstones of its accountability framework. The Commission suggests that indicators on reproductive, maternal and child health be disaggregated for equity considerations and that indicators be reported for social stratifiers such as wealth quintiles, gender, urban/rural residence and educational status in order to monitor progress towards the UN Secretary-General’s Global Strategy for Women’s and Children’s Health [8,9]. Moreover, countries have made equity a priority concern as stipulated in their health policies and strategic plans [10].

Bangladesh is one of the low-income countries that are on track for MDG 4 that is concerned with reducing child mortality. The under-five mortality rate stands at 48 per 1000 live births and by registering an average annual reduction rate (AARD) of more than 4.3%, the country is on track for achieving the MDG 4 target. An AARD of 4.3% is required to achieve the 4th MDG of reducing under-five mortality by two-thirds between 1990 and 2015 [11]. Similarly, with AARD of 5.9%, the country is on-track for achieving the MDG 5 target of reducing the maternal mortality ratio by 75% between 1990 and 2015 [12]. However, with estimated 7,200 maternal deaths each year, Bangladesh is considered as one of the seven countries accounting for 3%-5% of global maternal deaths each. These seven countries along with India, Nigeria and Afghanistan accounted for 60% of global maternal deaths in 2010 [12]. This suggests the need for intensified efforts to bring down the sheer magnitude of maternal deaths in the country with special focus on the poor and disadvantaged segment of society.

The objective of this study is to assess the magnitude of inequities in reproductive and maternal health services in Bangladesh using the 2007 Bangladesh demographic and health survey. MDG 5 aims at improving maternal health and is operationalized by two targets and six indicators as presented in Table 1[13].

**Table 1:** Official targets and indicators of MDG 5

**Target**	**Indicator**
5A: reduce by three-quarters, between 1990 and 2015, the maternal mortality ratio	5.1: maternal mortality ratio
	5.2: proportion of births attended by skilled health personnel
5B: achieve by 2015, universal access to reproductive health	5.3: contraceptive prevalence rate
	Indicator
	5.4: adolescent birth rate
	5.5: antenatal care coverage (at least 1 visit and at least 4 visits)
	5.6: unmet need for family planning

Although Bangladesh has already conducted DHS 2011 and produced a report, the raw data was not accessible to us at the time of writing this paper. Hence, it was not possible to base the analysis on the latest dataset. This is a limitation that has to be considered when reading this paper. However, it should also be noted that the data used in this study is not very outdated and the findings could be used in future studies that attempt to measure changes in inequity using the same methodology.

### Country profile: highlights

Bangladesh is a low-income country in South Asia. The gross national income per capita in 2011 was 1,529 (at constant 2005 PPP$). The country’s human development index (HDI) in the same period was 0.500 and ranked 146th out of 187 countries on the HDI scale. The inequality-adjusted HDI was, however, 0.363 – an overall loss of 27.4% in the human development index [14]. The share of income [15] of the richest 10% of the population is 35.9% compared to 2% for the poorest 10% of the population. The Gini coefficient, a measure of income inequality, is estimated at 0.458. Using upper and lower national poverty line estimates, the headcount ratio is estimated between 31.5% and 17.6% respectively [15].

According to the 2011 Population and Housing Census the country’s total population was about 149.8 million with a growth rate of 1.37%. With 1,015 persons per square kilometer, the country is one of the most densely populated countries in the world [16].

The health and development indicators have shown remarkable improvement over the years, placing the country among the few low-income countries that are on track for most of the health-related MDGs. Life expectancy at birth increased from 54 years in 1990 to 65 years in 2009 [11]. The under-five mortality rate decreased from 143 per 1,000 live births in 1990 to 48 per 1,000 live births in 2010 [17]. This achievement is registered despite critical health care resource constraints. Per capita expenditure on health in 2009 was only US$ 21 (at average exchange rate). Furthermore, physician and nurse/midwifery personnel densities per 10,000 population were 3.0 and 2.7 respectively [11].

The country has adopted the sector wide approach (SWAp) since 1998. The third SWAp programme (Health, population and nutrition sector development programme) covering the period 2011–2016 has been developed with the objective of improving access to services in order to improve morbidity and mortality, especially among women and children [18].

## Methods

### Data sources

Data from Bangladesh demographic and health survey (BDHS) 2007 is used for this analysis. The survey employed a two-stage stratified sampling technique to select 10,189 households in 361 primary sampling units (enumeration area or segment thereof). The survey aimed at obtaining 11,485 completed interviews with ever-married women age 10–49 years. Interviews were successfully completed in 10,400 households. Furthermore, 98.4% of the 11,178 eligible women completed the interview. Data was collected through questionnaire following MEASURE DHS model content and consisting of five modules: household questionnaire, women’s questionnaires, men’s questionnaire, community questionnaire and facility questionnaire [19].

### Variables and definitions

The reproductive and maternal health services analyzed in this study are defined in BDHS 2007 as indicated in Table 2.

**Table 2:** Reproductive and maternal health services included in the study and their definitions

**Service**	**Definition/measurement**
Antenatal care coverage	Percentage of women age 15–49 who had a live birth in the five years preceding the survey that received 4 or more antenatal visits
Components of antenatal care	Includes blood pressure, urine sample, blood sample, body weight, ultrasonography, iron tablets or syrup
Place of delivery	Percentage of live births in the five years preceding the survey delivered in a health facility (private or public) and percentage who delivered at home
Delivery by skilled health personnel	Percentage of births attended by doctors, nurses or midwives [25]
Delivery by untrained TBA	Percentage of births attended by *dais* (untrained traditional birth attendants)
Delivery by caesarean section	Percentage of live births during the five years preceding the survey delivered by caesarean section
Use of modern contraceptive	Percentage of currently married women age 15–49 using modern contraceptives

### Measurement of inequities

Equity in health is defined as the absence of systematic inequalities in health or in the major social determinants of health among people who have different positions in social hierarchy [20]. In measuring equity in health services three important steps need to be observed: (i) identification of the variable of interest whose distribution is to be measured; (ii) a measure of socio-economic status that classifies households or individuals into different socio-economic strata; and (iii) a measure of inequality.

The reproductive and maternal health services whose distribution we intend to measure are described in Table 1. We used the wealth decile generated from the wealth index factor score (variable v191 in BDHS 2007 raw data) as the socio-economic stratifier. Wealth decile 1 (D_1_) represents the poorest 10% of households and wealth decile 10 (D_10_) the richest 10%. The wealth index score in the BDHS 2007 was computed using information on ownership of household assets including ownership of durable goods and dwelling characteristics (such as source of drinking water and sanitation facilities) [19].

The most common complex measures that provide a summary measure of health inequality in a series of sub-groups with a natural ordering (ranking) are the slope (and relative) index of inequality and the concentration index [21].

The slope and relative indices of inequality were used in quantifying inequities in reproductive and maternal health services. These measures possess three important attributes of a good measure of equity: first, they reflect the experience of the entire population rather than two extreme groups only (D_1_ and D_10_); second, they take into account the socio-economic dimension of health inequalities; and third, they are sensitive to changes in the distribution of the population across socio-economic groups [22].

The slope index of inequality (SII) measures the absolute effect of changes in socio-economic status on the reproductive and maternal health service indicator of interest; while the relative index of inequality (RII) is a relative measure of the same. In other words, the SII and RII indicate the effect on utilization of reproductive and maternal health services when we move from the lowest to the highest socio-economic group, i.e. moving from D_1_ to D_10_.

Computing the SII and RII involves ranking the households from the lowest to the highest according to their socio-economic status (D_1_, D_2_, …, D_10_). Each wealth decile covers a range in the distribution of the population. The midpoint of this range in the cumulative distribution of the population is given as a rank to each wealth quintile. The SII is the slope of the regression line (Equation 1) showing the relationship between wealth decile’s use of reproductive and maternal health services and its relative socio-economic rank.

(1) $$y_{i}=\beta_{0}+\beta_{1}x_{i}+\epsilon$$

Where:

*y*_*i*_ = the value of the variable (reproductive and maternal health service use) of wealth decile *i*;

*x*_*i*_ = the relative rank of wealth decile *i*;

*β*_0_ = the constant term, captures the value of *y*_*i*_ when *x*_*i*_ equals zero;

*β*_1_ = the slope coefficient, indicates the amount of change in *y *as *x* changes by one unit; and

*ε* = the error term, captures the variation in *y *that cannot be explained by changes in *x*_*i.*_

The coefficient *β*_*i*_ represents the SII. The RII is derived from the SII according to the following:

(2) $$\mathrm{RII}=\frac{\mathrm{SII}}{\mu }=\frac{\beta_{i}}{\mu }$$

Where, *μ* is the mean value of the specific reproductive and maternal health service indicator.

However, as we have grouped the population into wealth deciles, the error term of the regression equation (*ε*) is heterskedastic rendering the ordinary Least Squares (OLS) estimates inefficient. To address this problem, the SII is estimated using the method of Weighted Least Squares (WLS) by running OLS regression on the following transformed equation [22]:

(3) $$y_{i}\sqrt{ni}=\beta_{0}\sqrt{n_{i}}+\beta_{1}x_{i}\sqrt{n_{i}}+\epsilon_{i}$$

Where, *n*_*i*_ is the number of individuals in each wealth decile.

It has to be noted that there is no constant term in equation (3). An example of the computation process is provided in Additional file 1.

The coefficient *β*_1_ shows the absolute difference in health status or health service use between the bottom and top of the wealth group distribution (i.e. difference between wealth Deciles 1 and 10) while taking into consideration the entire distribution of wealth.

The SII and RII have an advantage over simple measures such as the range, which compare the extreme groups only (Deciles 1 and 10 in this case) and pay no attention to the situation of the middle eight wealth deciles [21-23]. However, the SII and RII have limitations in that they are relatively more complex in their computation and require socio-economic subgroups with a hierarchical ranking [21,24].

Data was analyzed using STATA 10 statistical software and MS Excel.

## Results

### Descriptive statistics

Table 3 presents descriptive statistics of the reproductive and maternal health service indicators analyzed in this study. The decile ratios are also computed to assess the wealth-related inequalities between the two extremes and for comparison with the RII. Distribution of all variables by wealth decile is provided in (Additional file 2).

**Table 3:** Means and decile ratios of reproductive and maternal health services

**Service**	**Mean (%)**	**Decile ratio (D**_**10**_**/D**_**1**_**)**
Antenatal care – 1 visit	60.3	1.2
Antenatal care at least 4 visit	20.6	8.2
Antenatal care by a doctor	35.5	6.2
Antenatal care by a nurse	11.1	1.3
Antenatal care – no one	39.6	0.13
Ultrasonography	34.6	7.7
Weight taken	80.3	1.4
Blood pressure	86.4	1.1
Urine test	54.2	2.6
Iron	54.8	2.2
Delivery by skilled birth attendants	11.1	16.0
Delivery by a doctor	12.7	14.4
Delivery by a nurse/midwife	15.9	13.8
Delivery by untrained TBA	63.4	0.3
Delivery in a health facility	12.3	15.1
Delivery at home	85.1	0.43
Caesarean delivery	7.5	18.1
Use of modern contraception	44.9	1.2
FP message from radio	10.2	2.7

Focused antenatal care, that is at least four antenatal visits during the entire pregnancy, is remarkably low. There is a 40-percentage point drop when one compares focused antenatal care with ANC of less than 4 visits. Effective maternal health interventions may not be provided adequately when the number of ANC visits is less than the recommended minimum of four visits [25]. The decile ratio for four antenatal visits indicates that women from the richest 10% of households make eight times more visits as compared to those from the poorest 10%. However, for ANC of less than four visits, the inequalities are almost non-existent.

The percentage of women receiving antenatal care by doctors is more than three times that which is provided by nurses and midwives. Not unexpectedly, there is a high decile ratio indicating that most of the antenatal care provided by doctors accrues to the richest decile. On the other hand, although there is a 30% higher use of antenatal care by nurses and midwives by the wealthiest decile, the inequality gap is much narrower compared to antenatal care by doctors.

With respect to services provided during antenatal visits, the gap between the two deciles is glaringly high in ultrasonography – pregnant women from the wealthiest 10% households use ultrasonography about 8 times more than those from the least wealthy 10%. About half of the pregnant women only received iron supplementation and had their urine tested. Again, the wealthiest receive these two services more than the least wealthy (more than double).

**Figure 1:**
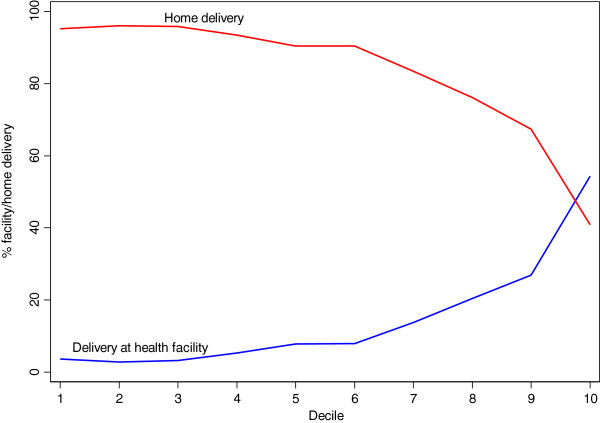
Delivery at home and in a health facility by wealth decile.

Delivery by skilled attendants is very low on average. Inequalities between the two wealth deciles are very high indicating that utilization by the wealthier groups is much higher than what is depicted by the aggregate mean. Delivery by untrained TBAs (63%) and home delivery (85%) demonstrate inequalities that are in “favour” of the poor. In other words, pregnant women from the least wealthy decile use untrained TBAs and home delivery 3.3 and 2.3 times more than the wealthiest 10%. Figure 1 depicts opposite trends in delivery at home and in a health facility by wealth decile.

Expectedly, delivery in health facility and delivery at home demonstrate diametrically opposite trends. While there is relatively higher rate of delivery in a health facility among the wealthiest, delivery at home is relatively lower.

Caesarean delivery, which is at 7.5%, is within accepted limits. However, the high decie ratio observed is indicative of the fact that there is both under-provision of the service in the poorest segment of society and over-provision in the wealthiest.

Use of modern family planning is 20% more among the wealthiest 10% compared to the least wealthy. Furthermore, the wealthiest 10% receive family planning messages from the radio more often that the poorest.

**Figure 2:**
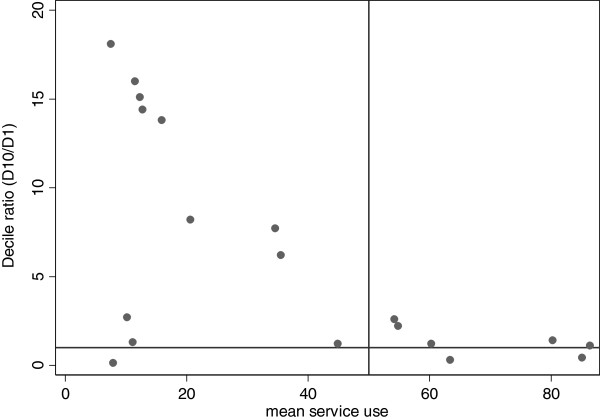
Mean service coverage vs. decile ratio.

It is also observed that lower mean coverage of services is more often associated with higher levels of inequality between the highest and lowest wealth deciles as depicted in Figure 2. As can be seen from Figure 2, the decile ratio is close to one (no inequality) in those services with more than 50% use rate as opposed to those where the mean is less than 50%.

### Inequities in reproductive and maternal health services

Even though the decile ratios are relatively easier to compute and understand, they only compare the two extreme groups and do not take into account inequalities in the middle eight deciles. As discussed previously, the slope and relative indices of inequality are used to remedy this problem.

The analysis indicates that with the exception of use of modern family planning, all other services under consideration in this study demonstrate wealth-related inequalities favouring the wealthiest segment of the population. Table 4 presents the SII and RII values and their 95% confidence intervals. The detailed regression outputs are provided in Additional file 3.

**Table 4:** Slope and relative indices of inequality, selected reproductive and maternal health services

		**95% CI**			**95% CI**	
**Service**	**SII**	**Lower**	**Upper**	**RII**	**Lower**	**Upper**
Antenatal care at least 4 visit	52.5	31.1	73.7	2.5	1.5	3.6
Antenatal care by a doctor	74.6	57.5	91.6	2.1	1.6	2.6
Antenatal care by a nurse	5.9	2.1	9.7	0.53	0.18	0.87
Antenatal care – no one	−59.3	−66.7	−52.0	−1.5	−1.7	−1.3
Ultrasonography	81.5	64.8	98.1	2.4	1.9	2.8
Weight taken	23.4	13.4	33.3	0.30	0.17	0.41
Blood pressure	14.7	7.8	21.6	0.17	0.09	0.25
Urine test	57.5	44.1	70.9	1.1	0.81	1.31
Iron	46.9	40.3	53.4	0.85	0.73	0.97
Delivery by skilled birth attendants	40.9	17.0	64.9	3.7	1.5	5.9
Delivery by a doctor	45.4	21.1	69.7	3.6	1.7	5.5
Delivery by a nurse/midwife	52.2	26.9	77.5	3.3	1.7	4.9
Delivery by untrained TBA	−44.8	−70.5	−19.1	−0.71	−1.1	−0.3
Delivery in a health facility	48.9	23.7	74.1	3.3	1.6	5.0
Delivery at home	−49.4	−74.3	−24.4	−0.58	−0.87	−0.29
Caesarean delivery	30.4	11.0	49.8	4.0	1.5	6.6
Use of modern contraception	5.0	−2.0	11.9	0.11	−0.04	0.27
FP message from radio	8.8	1.6	16.0	0.86	0.16	1.56

Antenatal care (at least four visits during the entire pregnancy) increases by more than 50 percentage points when moving from the least wealthy to the wealthiest pregnant women taking into consideration inequalities in all wealth deciles. It should be noted that a one unit change in relative rank indicates movement from the bottom wealth decile to the top most wealth decile.

The RII indicates that use of at least four antenatal visits among pregnant women from the wealthiest households is more than double that of the poorest ones. Antenatal care by a doctor increases by 75 percentage points when moving from the least wealthy decile to the wealthiest. In terms of the RII, the use of antenatal care by a doctor increases by more than double when moving from the least wealthy to the most wealthy decile and is statistically significant (P < 0.001). In contrast, antenatal care by a nurse increases by only 53%, signifying that although there is wealth-related inequality, the magnitude is less than that of antenatal care by a doctor.

The content of antenatal care provided is very important if the program is to contribute to better maternal and neonatal health outcomes and consequently achievement of MDGs 4 and 5 targets. The provision of ultrasonography, measurement of body weight, measurement of blood pressure, urine test and iron administration all indicate inequities favoring the wealthiest, albeit to differing degrees. Inequity in the provision of ultrasonography is the most pronounced one compared to the other services.

Skilled birth attendance (i.e. delivery by doctors, nurses or midwives) increases by 41 percentage points among the wealthiest as compared to the least wealthy (about four-fold). In line with expectation, delivery by untrained TBAs decreases by 45 percentage points among the wealthiest decile compared to the least wealthy. This implies that delivery by untrained personnel is most common among the least wealthy expectant women.

Delivery in a health facility shows inequity favouring the wealthiest – increasing by more than three-fold as compared to the least wealthy. On the other hand, home delivery decreases by 58% among the wealthiest 10%, implying that home delivery is more common among the poorest.

Caesarean delivery is most common among the wealthiest. Use of modern family planning does not exhibit any inequities (RII = 0.11; *p > 0.05*).

## Discussion and conclusions

This study examines wealth-related inequalities in reproductive and maternal health services using data from the Bangladesh demographic and health survey 2007. The slope and relative indices of inequality are used in quantifying the magnitude of inequities related to household wealth. Assessing inequities in reproductive and maternal health services is important to design interventions to increase access to the needed services and contribute to achievement of the relevant health-related MDG targets. Progress towards achievement of the MDG targets can be expedited if there is more focus on the poorest of society, as it is among the poorest groups that there is a significant potential for improvement [5].

Inequities in the services analyzed point to the need for an equity focus to make the gains in achieving the MDGs 4 and 5 targets all inclusive. Widespread inequities may potentially erode the achievements if they are not addressed through appropriate mult-sectoral interventions.

Non-use of antenatal care is more pronounced among the least wealthy women. Wealthy pregnant women more often receive antenatal care from doctors and nurses. Antenatal care is one of the pillars of the Safe Motherhood Initiative and helps provide interventions that are necessary for healthy outcomes of pregnancy [26]. Antenatal care creates a platform for reaching pregnant women with interventions that may be beneficial for their health and the outcomes of pregnancy. Receiving antenatal care at least four times, as recommended by WHO, increases the likelihood of receiving effective health promotion and preventive maternal health interventions during antenatal visits [25]. Antenatal care coverage is an indicator of the 5th Millenium Development Goal (MDG 5) related with improving maternal health [27]. The lack of the benefits of antenatal care among the least wealthy pregnant women might result in maternal health conditions that could have been identified and addressed early before potentially life-threatening conditions occur.

Pro-wealth inequality in the services provided in the antenatal program is of great concern, as it implies that the least wealthy pregnant women attending the program are not screened properly and given the necessary treatment in time. It is in particular worrying to see that examinations such as measurement of body weight and blood pressure, urine test and medicinal iron supplementation have a bias against the poorest. Non-administration of these services to less wealthy clients of the antenatal program would ultimately defeat the very objectives of the antenatal program and potentially discourage the future use of antenatal services.

Delivery by skilled attendants increases by almost four-fold when moving from the poorest to the wealthiest. The inequity is more or less the same when it is disaggregated by type of skilled provider – doctor or nurse/midwife. While the average figure for skilled attendance at birth shows a very low figure, disaggregation by wealth reveals that the situation among the poorest is of great concern and needs to be addressed vigorously through demand-enhancing and supply-side cost-effective measures if Bangladesh to achieve the MDG target. Skilled attendance at birth is the single most critical intervention for ensuring safe motherhood [28]. Delivery by untrained TBAs and home delivery are observed to significantly increase among the poorest compared to their rich counterparts. This does not bode well with improving maternal health among the poorest, as obstetric emergencies may not be addressed in a timely manner. All the necessary efforts should therefore be undertaken to ensure that deliveries are managed by skilled health workers in health facilities.

## Competing interests

The authors declare that they have no competing interests.

## Authors’ contributions

EZ designed the study, performed the analysis and drafted the report; YS, AA, LM, SKC and JK participated in the write-up and revision of the manuscript. All authors read and approved the final manuscript.

## Supplementary Material

Additional file 1 Example computation of the x-variable (relative rank) in the formula using the rate of caesarean section by wealth decile. Click here for file

Additional file 2 Distribution of indicators by wealth decile. Click here for file

Additional file 3 Regression output. Click here for file
