# Supplementary material for: Equity in reproductive and maternal health services in Bangladesh
Source: Int J Equity Health. 2013 Nov 14;12:90. doi: 10.1186/1475-9276-12-90 (PMC3842788; doi:10.1186/1475-9276-12-90)
Supplement: Additional file 1 — Example computation of the x-variable (relative rank) in the formula using the rate of caesarean section by wealth decile. [file 1475-9276-12-90-S1.docx]

**Additional file 1: Distribution of indicators by wealth decile**

| **Indicator** | **Decile** | **%** |
| --- | --- | --- |
| Antenatal care at least 4 visit | Poorest 10% | 7.7 |
|  | 2 | 8.6 |
|  | 3 | 7.8 |
|  | 4 | 13.5 |
|  | 5 | 14.2 |
|  | 6 | 17.3 |
|  | 7 | 24.2 |
|  | 8 | 31.0 |
|  | 9 | 36.3 |
|  | Wealthiest 10% | 62.6 |
|  | Mean | 20.6 |
|  | | |
| Antenatal care – 1 visit | Poorest 10% | 51.4 |
|  | 2 | 52.7 |
|  | 3 | 52.1 |
|  | 4 | 56.4 |
|  | 5 | 50.7 |
|  | 6 | 51.1 |
|  | 7 | 57.7 |
|  | 8 | 55.5 |
|  | 9 | 56.7 |
|  | Wealthiest 10% | 61.9 |
|  | Mean | 60.3 |
|  | | |
| Antenatal care by a doctor | Poorest 10% | 13.3 |
|  | 2 | 17.6 |
|  | 3 | 16.4 |
|  | 4 | 23.4 |
|  | 5 | 28.5 |
|  | 6 | 34.6 |
|  | 7 | 43.5 |
|  | 8 | 56.3 |
|  | 9 | 63.4 |
|  | Wealthiest 10% | 81.9 |
|  | Mean | 35.5 |
|  | | |
| Antenatal care by a nurse | Poorest 10% | 9.3 |
|  | 2 | 9.1 |
|  | 3 | 9.6 |
|  | 4 | 9.6 |
|  | 5 | 9.7 |
|  | 6 | 10.3 |
|  | 7 | 14.1 |
|  | 8 | 13.8 |
|  | 9 | 15.2 |
|  | Wealthiest 10% | 11.7 |
|  | Mean | 11.1 |

| **Indicator** | **Decile** | **%** |
| --- | --- | --- |
| Antenatal care – no one | Poorest 10% | 63.0 |
|  | 2 | 52.9 |
|  | 3 | 54.9 |
|  | 4 | 48.7 |
|  | 5 | 43.2 |
|  | 6 | 36.1 |
|  | 7 | 29.9 |
|  | 8 | 22.5 |
|  | 9 | 18.6 |
|  | Wealthiest 10% | 7.9 |
|  | Mean | 39.7 |
|  | | |
| Underwent Ultrasonography | Poorest 10% | 10.4 |
|  | 2 | 10.1 |
|  | 3 | 13.2 |
|  | 4 | 10.7 |
|  | 5 | 24.5 |
|  | 6 | 25.7 |
|  | 7 | 38.7 |
|  | 8 | 44.0 |
|  | 9 | 55.6 |
|  | Wealthiest 10% | 80.2 |
|  | Mean | 34.6 |
|  | | |
| Body weight taken | Poorest 10% | 68.8 |
|  | 2 | 79.8 |
|  | 3 | 72.5 |
|  | 4 | 74.9 |
|  | 5 | 77.5 |
|  | 6 | 78.5 |
|  | 7 | 76.2 |
|  | 8 | 86.4 |
|  | 9 | 84.5 |
|  | Wealthiest 10% | 94.5 |
|  | Mean | 80.3 |
|  | | |
| Blood pressure taken | Poorest 10% | 84.8 |
|  | 2 | 83.4 |
|  | 3 | 83.8 |
|  | 4 | 82.7 |
|  | 5 | 81.1 |
|  | 6 | 82.7 |
|  | 7 | 85.6 |
|  | 8 | 88.3 |
|  | 9 | 90.3 |
|  | Wealthiest 10% | 96.1 |
|  | Mean | 86.4 |

| **Indicator** | **Decile** | **%** |
| --- | --- | --- |
| Urine test | Poorest 10% | 33.2 |
|  | 2 | 38.5 |
|  | 3 | 36.6 |
|  | 4 | 37.4 |
|  | 5 | 50.2 |
|  | 6 | 45.8 |
|  | 7 | 58.9 |
|  | 8 | 66.1 |
|  | 9 | 64.4 |
|  | Wealthiest 10% | 85.9 |
|  | Mean | 54.2 |
|  | | |
| Iron tablets or syrup | Poorest 10% | 37.1 |
|  | 2 | 40.8 |
|  | 3 | 45.1 |
|  | 4 | 48.9 |
|  | 5 | 55.0 |
|  | 6 | 54.4 |
|  | 7 | 62.5 |
|  | 8 | 68.1 |
|  | 9 | 69.3 |
|  | Wealthiest 10% | 81.9 |
|  | Mean | 54.8 |
|  | | |
| Delivery by skilled birth attendants | Poorest 10% | 3.1 |
|  | 2 | 2.6 |
|  | 3 | 2.5 |
|  | 4 | 3.5 |
|  | 5 | 7.0 |
|  | 6 | 6.2 |
|  | 7 | 10.7 |
|  | 8 | 17.1 |
|  | 9 | 24.8 |
|  | Wealthiest 10% | 51.0 |
|  | Mean | 11.1 |
|  | | |
| Delivery by a doctor | Poorest 10% | 3.8 |
|  | 2 | 2.7 |
|  | 3 | 3.0 |
|  | 4 | 4.3 |
|  | 5 | 7.8 |
|  | 6 | 7.6 |
|  | 7 | 13.0 |
|  | 8 | 21.0 |
|  | 9 | 28.7 |
|  | Wealthiest 10% | 54.5 |
|  | Mean | 12.7 |

| **Indicator** | **Decile** | **%** |
| --- | --- | --- |
| Delivery by a nurse/midwife | Poorest 10% | 4.5 |
|  | 2 | 3.9 |
|  | 3 | 4.4 |
|  | 4 | 6.6 |
|  | 5 | 10.8 |
|  | 6 | 11.9 |
|  | 7 | 17.0 |
|  | 8 | 25.4 |
|  | 9 | 35.6 |
|  | Wealthiest 10% | 61.8 |
|  | Mean | 15.9 |
|  | | |
| Delivery by untrained TBA | Poorest 10% | 72.0 |
|  | 2 | 71.6 |
|  | 3 | 71.2 |
|  | 4 | 73.0 |
|  | 5 | 74.5 |
|  | 6 | 69.2 |
|  | 7 | 59.5 |
|  | 8 | 55.3 |
|  | 9 | 47.1 |
|  | Wealthiest 10% | 22.0 |
|  | Mean | 63.4 |
|  | | |
| Delivery in a health facility | Poorest 10% | 3.6 |
|  | 2 | 2.8 |
|  | 3 | 3.2 |
|  | 4 | 5.2 |
|  | 5 | 7.8 |
|  | 6 | 7.9 |
|  | 7 | 13.7 |
|  | 8 | 20.4 |
|  | 9 | 26.9 |
|  | Wealthiest 10% | 54.3 |
|  | Mean | 12.3 |
|  | | |
| delivery at home | Poorest 10% | 95.3 |
|  | 2 | 96.1 |
|  | 3 | 95.9 |
|  | 4 | 93.4 |
|  | 5 | 90.5 |
|  | 6 | 90.5 |
|  | 7 | 83.5 |
|  | 8 | 76.2 |
|  | 9 | 67.3 |
|  | Wealthiest 10% | 40.9 |
|  | Mean | 85.1 |

| **Indicator** | **Decile** | **%** |
| --- | --- | --- |
| Caesarean delivery | Poorest 10% | 2.1 |
|  | 2 | 1.2 |
|  | 3 | 1.3 |
|  | 4 | 2.7 |
|  | 5 | 3.0 |
|  | 6 | 4.0 |
|  | 7 | 6.2 |
|  | 8 | 13.3 |
|  | 9 | 16.2 |
|  | Wealthiest 10% | 38.8 |
|  | Mean | 7.5 |
|  | | |
| Use of modern contraception | Poorest 10% | 42.2 |
|  | 2 | 45.4 |
|  | 3 | 41.8 |
|  | 4 | 48.8 |
|  | 5 | 42.3 |
|  | 6 | 41.4 |
|  | 7 | 47.6 |
|  | 8 | 46.7 |
|  | 9 | 45.1 |
|  | Wealthiest 10% | 49.3 |
|  | Mean | 44.9 |
|  | | |
| Message on family planning from radio | Poorest 10% | 3.6 |
|  | 2 | 4.9 |
|  | 3 | 9.4 |
|  | 4 | 10.4 |
|  | 5 | 12.7 |
|  | 6 | 12.9 |
|  | 7 | 14.3 |
|  | 8 | 15.0 |
|  | 9 | 11.9 |
|  | Wealthiest 10% | 9.8 |
|  | Mean | 10.3 |
