# Supplementary material for: Equity in reproductive and maternal health services in Bangladesh
Source: Int J Equity Health. 2013 Nov 14;12:90. doi: 10.1186/1475-9276-12-90 (PMC3842788; doi:10.1186/1475-9276-12-90)
Supplement: Additional file 2 — Distribution of indicators by wealth decile. [file 1475-9276-12-90-S2.docx]

**Additional file 2**

**Example computation of the x-variable (relative rank) in the formula using the rate of caesarean section by wealth decile.**

| Wealth decile | CS rate | Freq. | % | Cumm.% | range | Midpoint (x-variable) |
| --- | --- | --- | --- | --- | --- | --- |
| 1 | 0.021 (or 2.1%) | 675 | 10.98 | 10.98 | 0-10.98 | 0.054 |
| 2 | 0.012 | 624 | 10.15 | 21.13 | 10.98-21.13 | 0.161 |
| 3 | 0.013 | 664 | 10.80 | 31.93 | 21.3-31.93 | 0.265 |
| 4 | 0.027 | 642 | 10.44 | 42.37 | 31.93-42.37 | 0.372 |
| 5 | 0.030 | 578 | 9.40 | 51.77 | 42.37-51.77 | 0.471 |
| 6 | 0.040 | 585 | 9.52 | 61.29 | 51.77-61.29 | 0.565 |
| 7 | 0.062 | 598 | 9.73 | 71.01 | 61.29-71.01 | 0.662 |
| 8 | 0.133 | 564 | 9.17 | 80.19 | 71.01-80.19 | 0.756 |
| 9 | 0.162 | 608 | 9.89 | 90.08 | 80.19-90.08 | 0.851 |
| 10 | 0.388 (or 38.8%) | 610 | 9.92 | 100.00 | 90.08-100.0 | 0.950 |
| Total | 0.075 | 6148 | 100.0 |  |  |  |

The x-variable (the relative rank) is the mid-point of the range in the cumulative distribution of the population. For example, the proportion of women in the first decile (caesarean section rate of 2.1%) was 10.98% (cumulative %=10.98); and those in the second decile (caesarean section rate of 1.2%) was 10.15% (cumm. % 21.13). The value of *x* for the first decile is 0.054 [i.e. (0+10.98)/2=5.4%, which is 0.054]. Similarly for Decile 2, it is 0.161 [(10.98+21.13)/2=16.1%=0.161].
