# Supplementary material for: Equity in reproductive and maternal health services in Bangladesh
Source: Int J Equity Health. 2013 Nov 14;12:90. doi: 10.1186/1475-9276-12-90 (PMC3842788; doi:10.1186/1475-9276-12-90)
Supplement: Additional file 3 — Regression output. [file 1475-9276-12-90-S3.pdf]

Additional material – regression output

. Antenatal care at least 4 visit

Source	SS	df	MS		
				Number of obs =	10
				F(2, 8) =	52.69
Model	364.14274	2	182.07137	Prob > F =	0.0000
Residual	27.646102	8	3.45576275	R-squared =	0.9294
				Adj R-squared =	0.9118
Total	391.788842	10	39.1788842	Root MSE =	1.859

ANC	Coef.	Std. Err.	t	P>|t|	[95% Conf.	Interval]
sqrtn	-.0357977	.0532076	-0.67	0.520	-.1584946	.0868993
rank1	.5246172	.0922738	5.69	0.000	.3118335	.737401

. Antenatal care by a doctor

Source	SS	df	MS		
				Number of obs =	10
				F(2, 8) =	212.90
Model	944.842352	2	472.421176	Prob > F =	0.0000
Residual	17.7520711	8	2.21900889	R-squared =	0.9816
				Adj R-squared =	0.9769
Total	962.594423	10	96.2594423	Root MSE =	1.4896

ANC by Dr	Coef.	Std. Err.	t	P>|t|	[95% Conf.	Interval]
sqrtn	.0094854	.0426256	0.22	0.829	-.0888093	.1077802
rank1	.7455247	.073923	10.09	0.000	.575058	.9159913

. **Antenatal care by nurse**

```
      Source |       SS       df       MS              Number of obs =      10
-------------+------------------------------           F(  2,     8) =  283.73
       Model |  63.6736286     2  31.8368143           Prob > F      =  0.0000
    Residual |  .897663239     8  .112207905           R-squared     =  0.9861
-------------+------------------------------           Adj R-squared =  0.9826
       Total |  64.5712918    10  6.45712918           Root MSE      =  .33497

------------------------------------------------------------------------------
ANC by nurse |      Coef.   Std. Err.      t    P>|t|     [95% Conf. Interval]
-------------+----------------------------------------------------------------
       sqrtn |   .0829427   .0095852     8.65   0.000     .0608391    .1050463
       rank  |   .0590499   .0166231     3.55   0.007      .020717    .0973828
------------------------------------------------------------------------------
```

. **Antenatal care – no one**

```
      Source |       SS       df       MS              Number of obs =      10
-------------+------------------------------           F(  2,     8) = 1028.27
       Model |  839.266343     2  419.633171           Prob > F      =  0.0000
    Residual |   3.2647755     8  .408096937           R-squared     =  0.9961
-------------+------------------------------           Adj R-squared =  0.9952
       Total |  842.531118    10  84.2531118           Root MSE      =  .63882

------------------------------------------------------------------------------
   ancnowan1 |      Coef.   Std. Err.      t    P>|t|     [95% Conf. Interval]
-------------+----------------------------------------------------------------
       sqrtn |   .6728553   .0182798    36.81   0.000     .6307019    .7150086
       rank  |  -.5935216   .0317016   -18.72   0.000    -.6666256   -.5204175
------------------------------------------------------------------------------
```

. **Ultrasonography**

Source	SS	df	MS
Model	606.203052	2	303.101526
Residual	10.5199714	8	1.31499643
Total	616.723023	10	61.6723023

Number of obs =	10
F(2, 8) =	230.50
Prob > F =	0.0000
R-squared =	0.9829
Adj R-squared =	0.9787
Root MSE =	1.1467

ultrasound	Coef.	Std. Err.	t	P>|t|	[95% Conf. Interval]	
sqrtn	-.0290398	.0416343	-0.70	0.505	-.1250487	.0669691
rank	.8149368	.0722278	11.28	0.000	.6483793	.9814943

. **Body weight**

Source	SS	df	MS
Model	2036.89599	2	1018.44799
Residual	3.72772119	8	.465965149
Total	2040.62371	10	204.062371

Number of obs =	10
F(2, 8) =	2185.67
Prob > F =	0.0000
R-squared =	0.9982
Adj R-squared =	0.9977
Root MSE =	.68262

Weight	Coef.	Std. Err.	t	P>|t|	[95% Conf. Interval]	
sqrtn	.6958857	.0247895	28.07	0.000	.638721	.7530504
rank	.2336623	.0430065	5.43	0.001	.1344892	.3328354

. Blood pressure

```
      Source |       SS       df       MS              Number of obs =      10
-------------+------------------------------           F(  2,     8) = 5121.41
       Model |  2326.56031     2  1163.28016           Prob > F      =  0.0000
    Residual |  1.81712359     8  .227140448           R-squared     =  0.9992
-------------+------------------------------           Adj R-squared =  0.9990
       Total |  2328.37744    10  232.837744           Root MSE      =  .47659

------------------------------------------------------------------------------
         BP  |      Coef.   Std. Err.      t    P>|t|     [95% Conf. Interval]
-------------+----------------------------------------------------------------
       sqrtn |    .796822   .0173036    46.05   0.000     .7569199    .8367242
       rank  |   .1470914   .0300185     4.90   0.001     .0778686    .2163142
------------------------------------------------------------------------------
```

. Urine test

```
      Source |       SS       df       MS              Number of obs =      10
-------------+------------------------------           F(  2,     8) =  622.96
       Model |  1056.24092     2  528.120458           Prob > F      =  0.0000
    Residual |   6.7821263     8  .847765788           R-squared     =  0.9936
-------------+------------------------------           Adj R-squared =  0.9920
       Total |  1063.02304    10  106.302304           Root MSE      =  .92074

------------------------------------------------------------------------------
  Urine test |      Coef.   Std. Err.      t    P>|t|     [95% Conf. Interval]
-------------+----------------------------------------------------------------
       sqrtn |    .275965   .0334375     8.25   0.000     .1988579    .3530721
       rank  |   .5752264   .0580095     9.92   0.000     .4414563    .7089966
------------------------------------------------------------------------------
```

. Iron supplementation

```
     Source |       SS       df       MS              Number of obs =      10
-------------+------------------------------           F(  2,     8) = 2526.17
       Model |  1658.79034     2   829.39517           Prob > F      =  0.0000
    Residual |  2.62656642     8  .328320803           R-squared     =  0.9984
-------------+------------------------------           Adj R-squared =  0.9980
       Total |  1661.41691    10  166.141691           Root MSE      =  .57299

------------------------------------------------------------------------------
       Iron  |      Coef.   Std. Err.      t    P>|t|     [95% Conf. Interval]
-------------+----------------------------------------------------------------
       sqrtn |   .3302095   .0163955    20.14   0.000     .2924015    .3680175
       rank  |   .4688557   .0284339    16.49   0.000      .403287    .5344245
------------------------------------------------------------------------------
```

. Delivery by skilled birth attendants

```
     Source |       SS       df       MS              Number of obs =      10
-------------+------------------------------           F(  2,     8) =   16.71
       Model |  182.849468     2   91.424734           Prob > F      =  0.0014
    Residual |  43.7581419     8  5.46976773           R-squared     =  0.8069
-------------+------------------------------           Adj R-squared =  0.7586
       Total |   226.60761    10   22.660761           Root MSE      =  2.3388

------------------------------------------------------------------------------
      SBA    |      Coef.   Std. Err.      t    P>|t|     [95% Conf. Interval]
-------------+----------------------------------------------------------------
       sqrtn |  -.0784821   .0599076    -1.31   0.227    -.2166293    .0596651
       rank  |    .409441   .1038938     3.94   0.004     .1698614    .6490205
------------------------------------------------------------------------------
```

. Delivery by a doctor

```
     Source |       SS       df       MS              Number of obs =      10
-------------+------------------------------           F(  2,     8) =   20.52
       Model |  231.401394     2  115.700697           Prob > F      =  0.0007
    Residual |   45.115035     8  5.63937937           R-squared     =  0.8368
-------------+------------------------------           Adj R-squared =  0.7961
       Total |  276.516429    10  27.6516429           Root MSE      =  2.3747

------------------------------------------------------------------------------
  Del doctor |      Coef.   Std. Err.      t    P>|t|     [95% Conf. Interval]
-------------+----------------------------------------------------------------
       sqrtn |  -.0833378   .0608294    -1.37   0.208    -.2236106    .0569349
       rank  |   .4541238   .1054923     4.30   0.003      .210858    .6973895
------------------------------------------------------------------------------
```

. Delivery by a nurse

```
      Source |       SS       df       MS              Number of obs =      10
-------------+------------------------------           F(  2,     8) =   26.89
       Model |  321.948445     2  160.974222           Prob > F      =  0.0003
    Residual |  47.8880426     8  5.98600533           R-squared     =  0.8705
-------------+------------------------------           Adj R-squared =  0.8381
       Total |  369.836488    10  36.9836488           Root MSE      =  2.4466

------------------------------------------------------------------------------
   Del nurse |      Coef.   Std. Err.      t    P>|t|     [95% Conf. Interval]
-------------+----------------------------------------------------------------
       sqrtn |  -.0848997   .0632684    -1.34   0.216    -.2307968    .0609974
       rank  |   .5217388   .1097253     4.75   0.001     .2687117    .7747659
------------------------------------------------------------------------------
```

. Delivery by untrained attendant

```
     Source |       SS       df       MS              Number of obs =      10
-------------+------------------------------           F(  2,     8) =  193.94
       Model |  2444.54641     2   1222.2732           Prob > F      =  0.0000
    Residual |  50.4197292     8  6.30246614           R-squared     =  0.9798
-------------+------------------------------           Adj R-squared =  0.9747
       Total |  2494.96614    10  249.496614           Root MSE      =  2.5105

------------------------------------------------------------------------------
Del untrained|      Coef.   Std. Err.      t    P>|t|     [95% Conf. Interval]
-------------+----------------------------------------------------------------
       sqrtn |   .8415929   .0643062    13.09   0.000     .6933025    .9898833
       rank |   -.448065    .111522    -4.02   0.004    -.7052352   -.1908948
------------------------------------------------------------------------------
```

. Delivery in health facility

```
     Source |       SS       df       MS              Number of obs =      10
-------------+------------------------------           F(  2,     8) =   19.36
       Model |  203.629175     2  101.814587           Prob > F      =  0.0009
    Residual |  42.0659002     8  5.25823752           R-squared     =  0.8288
-------------+------------------------------           Adj R-squared =  0.7860
       Total |  245.695075    10  24.5695075           Root MSE      =  2.2931
------------------------------------------------------------------------------
Del facility |      Coef.   Std. Err.      t    P>|t|     [95% Conf. Interval]
-------------+----------------------------------------------------------------
       sqrtn |  -.0759783   .0597914    -1.27   0.240    -.2138577     .061901
       rank  |    .428894    .103696     4.14   0.003     .1897707    .6680173
```

. Delivery at home

```
      Source |       SS       df       MS              Number of obs =      10
-------------+------------------------------           F(  2,     8) =  369.08
       Model |  4382.35386     2  2191.17693           Prob > F      =  0.0000
    Residual |  47.4944944     8   5.9368118           R-squared     =  0.9893
-------------+------------------------------           Adj R-squared =  0.9866
       Total |  4429.84835    10  442.984835           Root MSE      =  2.4366

------------------------------------------------------------------------------
   Del home1 |      Coef.   Std. Err.      t    P>|t|     [95% Conf. Interval]
-------------+----------------------------------------------------------------
       sqrtn |   1.079449   .0624032    17.30   0.000     .9355468    1.223351
       rank  |  -.4939242   .1082225    -4.56   0.002    -.7434857   -.2443626
------------------------------------------------------------------------------
```

. Caesarean delivery

```
      Source |       SS       df       MS              Number of obs =      10
-------------+------------------------------           F(  2,     8) =   13.00
       Model |  93.5916251     2  46.7958125           Prob > F      =  0.0031
    Residual |  28.8029799     8  3.60037249           R-squared     =  0.7647
-------------+------------------------------           Adj R-squared =  0.7058
       Total |  122.394605    10  12.2394605           Root MSE      =  1.8975

------------------------------------------------------------------------------
          CS |      Coef.   Std. Err.      t    P>|t|     [95% Conf. Interval]
-------------+----------------------------------------------------------------
       sqrtn |  -.0647189   .0485839    -1.33   0.220    -.1767536    .0473158
       rank |   .3038665   .0842565     3.61   0.007     .1095708    .4981623
------------------------------------------------------------------------------
```

. FP use

```
      Source |       SS       df       MS              Number of obs =      10
-------------+------------------------------           F(  2,     8) = 1355.06
       Model |  6208.49988     2  3104.24994           Prob > F      =  0.0000
    Residual |  18.3268793     8  2.29085991           R-squared     =  0.9971
-------------+------------------------------           Adj R-squared =  0.9963
       Total |  6226.82676    10  622.682676           Root MSE      =  1.5136

------------------------------------------------------------------------------
      FP use |      Coef.   Std. Err.      t    P>|t|     [95% Conf. Interval]
-------------+----------------------------------------------------------------
       sqrtn |   .4258783   .0173916    24.49   0.000     .3857732    .4659835
       rank1 |   .0497383   .0301613     1.65   0.138    -.0198137    .1192903
------------------------------------------------------------------------------
```

. FP message from radio

```
      Source |       SS       df       MS              Number of obs =      10
-------------+------------------------------           F(  2,     8) =   72.77
       Model |  355.198675     2  177.599338           Prob > F      =  0.0000
    Residual |  19.5253566     8  2.44066958           R-squared     =  0.9479
-------------+------------------------------           Adj R-squared =  0.9349
       Total |  374.724032    10  37.4724032           Root MSE      =  1.5623

------------------------------------------------------------------------------
      Radio  |      Coef.   Std. Err.      t    P>|t|     [95% Conf. Interval]
-------------+----------------------------------------------------------------
       sqrtn |   .0609644   .0179513     3.40   0.009     .0195687    .1023601
       rank1 |    .087826   .0311318     2.82   0.022     .0160358    .1596161
------------------------------------------------------------------------------
```
